# Aftermath of Apixaban: Atypical Anticoagulation Aftereffect

**DOI:** 10.1155/2022/6221640

**Published:** 2022-10-22

**Authors:** Bhesh Karki, Louis Costanzo, Sameer Joshi, Aleksey Fiksman

**Affiliations:** ^1^Department of Internal Medicine, SUNY Downstate Health Sciences University, Brooklyn 11203, NY, USA; ^2^Department of Internal Medicine, Trumbull Regional Medical Center, Warren 44483, OH, USA; ^3^Brooklyn VA Medical Center, 800 Poly Pl, Brooklyn 11209, NY, USA

## Abstract

An elderly man with prostate cancer and a deep vein thrombosis (DVT) of the lower extremity diagnosed 12 days ago on apixaban presented with a new-onset palpable rash on both of his legs. Extensive laboratory workup was largely unremarkable, except for multiple skin punch biopsies revealing deposition of immunoglobulin A (IgA) in the superficial blood vessels with infiltration of leukocytes, concerning for a small vessel vasculitis. Given the temporal association along with the negative workup, the rash was attributed to apixaban, and the anticoagulation regimen was switched to dabigatran. At a 2-week follow-up visit, the patient was asymptomatic and tolerating dabigatran without any adverse events.

## 1. Introduction

The 2012 Chapel Hill Consensus Conference (CHCC) defines cutaneous small vessel vasculitis as a single-organ vasculitis that involves small arteries in the skin. [1] It is often referred to as hypersensitivity vasculitis or leukocytoclastic vasculitis (LCV) and is characterized with neutrophils as the major inflammatory infiltrate. Apixaban is a reversible and direct inhibitor of factor Xa. It is an uncommon cause of LCV. There have been very few reported cases of this unusual condition. [2–4] We report a case of an elderly male who presented with palpable purpura shortly after the initiation of apixaban for a DVT and was diagnosed with cutaneous leukocytoclastic vasculitis. This case thus draws attention to an uncommon, but potential adverse reaction of apixaban.

## 2. Case Description

A 72-year-old man with prostate cancer on androgen deprivation therapy (monthly leuprolide injections) and likely a provoked deep vein thrombosis (DVT) of the left lower extremity (diagnosed 12 days ago) secondary to the cancer presented to the emergency department with complaints of a rash on his bilateral lower legs which he noticed 1 week ago. He described the rash as red, nonpruritic, mildly painful, and raised. The rash started near his ankles and spread rapidly up his legs to his buttocks. He denied any fever, joint pain, abdominal pain, or changes in urinary habits. He denied any recent travel or experiencing this type of rash before.

His other past medical history included hypertension on losartan, type 2 diabetes on insulin, and chronic kidney disease. His only new medication was apixaban 5mg twice daily that he was taking for 12 days for the recently diagnosed DVT. He had completed a seven day course of 10mg of apixaban twice daily and was taking 5mg twice daily on the day of presentation. He had never taken any anticoagulant prior to this and had no known drug allergies or other recent medication changes.

On examination, his vitals and systemic exam were unremarkable. Skin exam revealed diffuse palpable tender nonblanching erythematous patches extending from the dorsum of both feet bilaterally to the thigh with few scattered lesions on the buttocks. A fundoscopic eye examination was unremarkable, and there were no other lesions noted on his body. An extensive laboratory workup was performed (see Tables 1 and 2) and a chest X-ray was without any significant findings. The urinalysis results revealed specific gravity-1.011, blood cell-11/hpf, hyaline casts-3/hpf, and granular cast 1/hpf. Urine and blood cultures were without any growth.

Both rheumatology and dermatology were consulted, and a skin biopsy was recommended for further evaluation. The 3 skin punch biopsies obtained (2 from left lateral thigh and 1 from right lower extremity) revealed perivascular infiltration of neutrophils within vessels in the upper dermis with leukocytoclastia, or neutrophilic debris. A direct immunofluorescence study showed intense granular vessel wall staining with IgA consistent with leukocytoclastic vasculitis.

Apixaban was discontinued on the day of admission, and he was anticoagulated with low-molecular-weight-heparin (lovenox). His other home medications including insulin were continued. His pain was managed with acetaminophen and moisturizer was applied to his legs bilaterally. Upon discharge, he was transitioned from lovenox to dabigatran. At his 2-week follow-up visit, he had minimal cutaneous involvement, but no discomfort. At 1-month follow-up, the skin lesions had greatly resolved with near complete resolution.

## 3. Discussion

The common etiologies of the palpable purpura like infectious (no signs of sepsis, normal leukocyte count, and negative blood culture), hematologic/coagulopathic (normal platelet, prothrombin time, and partial thromboplastin time), autoimmune/rheumatologic/systemic vasculitis (negative RF, anti-CCP, ANA, ANCA, normal urinary sediment, slightly elevated complement, and normal eosinophil) were ruled out. Although rare, LCV can present as an extraintestinal manifestation of ulcerative colitis, however, more than 2/3^rd^ patients have active intestinal disease at the time of diagnosis. [5] This patient did not have any gastrointestinal complaints. A definitive cause of palpable purpura is not identified in about 50% cases of cutaneous small vessel vasculitis. [6] However, a drug reaction is a common etiology for leukocytoclastic vasculitis [7] and about 10–15% cases of cutaneous vasculitis are drug induced. [8] Some medications documented to induce LCV are clozapine, antibiotics like ciprofloxacin, vancomycin, clomiphene, warfarin, and rivaroxaban. [9–13] Our patient scored a 6 using the Naranjo scale questionnaire indicating a likely adverse drug reaction. Cases of LCV have been documented with leuprolide; however, these usually occur after the second dose. [14] He had been on leuprolide for nearly 2 years making this drug an unlikely cause of the rash. The only new medication for him was apixaban. Given the temporal association and few prior documented reports along with the Naranjo score, the rash was ultimately attributed to apixaban.

**Figure 1:**
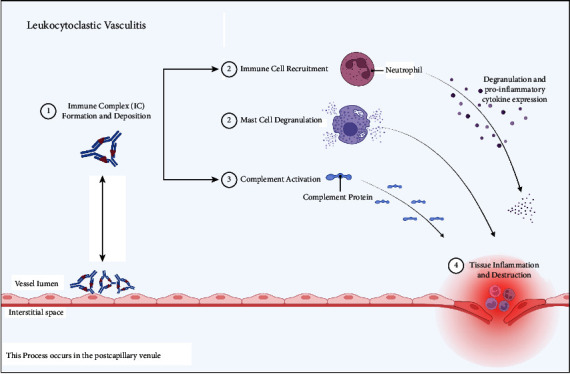
Pathophysiology of leukocytoclastic vasculitis. Immune complexes are deposited within the vessel wall. These complexes activate immune cells, mast cells, and complement proteins. This eventually leads to tissue inflammation and vessel damage. Figure “Created with BioRender.com”.

The inflammatory cascade in LCV mainly occurs in postcapillary venules, where the neutrophils undergo leukocytoclasis, meaning destruction and release of nuclear debris that incite the inflammation and damage of vessels (see Figure 1). [15] While it is possible for a drug-induced LCV to evolve into a systemic vasculitis, this is a rare complication. [16]

The literature related to apixaban-induced LCV reveals cases with comparable clinical presentations, histopathologic findings, and a relatively parallel clinical course with nearly identical outcomes. The onset of cutaneous symptoms has been previously documented in 10–28 days after initiating therapy with apixaban. [2–4] Our patient noticed symptoms after approximately 7 days of apixaban. The presentation of palpable purpuric lesions on the lower extremities and buttocks without any systemic involvement is like those seen in the prior cases. [2–4] Also, the major histologic finding in these cases is perivascular infiltration of neutrophils within vessels in the dermis with or without the immunoglobulin deposition. [2–4]

The treatment for LCV is mainly supportive, directed towards the underlying cause, and involves cessation of the offending agent. Steroids can be considered if the disease is severe, as reported in a patient who had complete resolution of symptoms after a 5-week oral prednisone course. [3] In the prior cases, cessation of apixaban resulted in a fairly rapid resolution of symptoms (about 3–12 weeks) with a favorable outcome. [2–4] To add to the literature, our patient underwent near complete resolution of symptoms at approximately 6 weeks.

A special mention is the possible cross reactivity of direct oral anticoagulants (DOACs). Anis and Jandreau reported the persistence of rash in a protein C deficient patient when switching from apixaban to rivaroxaban; which eventually subsided within 24 hours of starting warfarin. [17] Similarly, Isaq et al. reported a second episode of purpuric rash in a patient on apixaban, with the first episode of the similar rash while on rivaroxaban. [18] Our patient was switched to a DOAC with a different mechanism: a direct thrombin inhibitor, dabigatran. It is important to consider the potentiality of toxicity when switching between DOACs; specifically, those with similar structures and mechanisms of action.

## Figures and Tables

**Table 1:** Laboratory workup. Significant abnormal values include elevation in creatinine consistent with chronic kidney disease and elevated inflammatory markers.

Lab test	Value	Reference range
White blood cell	6.2 K/uL	4.5–11 K/uL
Hemoglobin	12.0 g/dL	13–18 g/dL
Platelet	275 K/uL	150–450 K/uL
Urea nitrogen	17 mg/dl	6–22 mg/dL
Creatinine	1.7 mg/dl	0.4–1.2 mg/dL
Protein, total	6.3 g/dl	6.4–8.2 mg/dL
Albumin	3.6 g/dl	3.8–5.1 mg/dL
Bilirubin, total	0.6 mg/dl	0.1–1.2 mg/dL
Alkaline phosphatase	82 U/L	42–121 U/L
Aspartate transaminase	18 U/L	10–42 U/L
Alanine transaminase	18 U/L	10–40 U/L
Glomerular filtration rate	42 ml/min	≥60 ml/min
PT/PTT	12.8 s/26.2 s	9.6–12.4 s/28–36.3 s
Erythrocyte sedimentation rate	65 mm/hr	0–15 mm/hr
C-reactive protein	0.74 mg/dl	0–0.5 mg/dl
Antistreptolysin O	22.9 IU/mL	0–200 IU/mL

**Table 2:** Additional laboratory testing ordered.

Lab test	Value	Reference range
Hepatitis C ab	Negative	Negative
Rheumatoid factor	<3.5 IU/mL	0–39 IU/mL
CCP antibodies IgG/IgA	5 units	0–19 units
Antinuclear antibodies	Negative	<1 : 80
Cytoplasmic ANCA	<1 : 20 titer	<1 : 20
Perinuclear ANCA	<1 : 20 titer	<1 : 20
Myeloperoxidase Ab	<9 U/mL	0.0–9 U/mL
Proteinase-3 Ab	<3.5 U/mL	0.0–3.5 U/mL
Atypical pANCA	<1 : 20 titer	<1 : 20
Complement C3	231.8 mg/dL	90–180 mg/dL
Complement C4	44.9 mg/dL	9–36 mg/dL
Cardiolipin IgM ab	<9 U/mL	0–12 U/mL
Cardiolipin IgG ab	<9 U/mL	0–14 U/mL
Cardiolipin IgA ab	<9 U/mL	0–11 U/mL
Cryoglobulin	Not detected	None
Beta-2 glycoprotein I IgG ab	<9 units	0–20 units
Beta-2 glycoprotein I Ig M ab	15 units	0–25 units
Beta-2 glycoprotein I Ig A ab	15 units	0–32 units
